# HPV 16 Is Related to the Progression of Cervical Intraepithelial Neoplasia Grade 2: A Case Series

**DOI:** 10.1155/2013/328909

**Published:** 2013-12-04

**Authors:** Maria Gabriela Loffredo D'Ottaviano, Michelle Garcia Discacciati, Maria Antonieta Andreoli, Maria Cecília Costa, Lara Termini, Silvia H. Rabelo-Santos, Luisa Lina Villa, Luiz Carlos Zeferino

**Affiliations:** ^1^School of Medical Sciences, State University of Campinas (UNICAMP), 101 Rua Alexander Fleming, 13083-881 Campinas, SP, Brazil; ^2^School of Pharmaceutical Sciences, University of São Paulo (USP), Rua Prof. Lineu Prestes, 580, 05508-000 São Paulo, SP, Brazil; ^3^School of Medicine, Santa Casa de São Paulo, INCT-HPV, Rua Marquês de Itú, 381, 01223-001 São Paulo, SP, Brazil; ^4^School of Pharmacy, Federal University of Goiás, Avenida Universitária, 74605-220 Goiânia, GO, Brazil

## Abstract

*Purpose*. To describe the acquisition, persistence, and clearance of HPV infection in women with CIN 2 followed up for 12 months. *Methods*. Thirty-seven women with CIN 2 biopsy, who have proven referral to cervical smear showing low-grade squamous intraepithelial lesions or atypical squamous cells of undetermined significance and tested for HPV, were followed up for one year with cervical smear, colposcopy, and HPV test every three months. HPV DNA was detected by the polymerase chain reaction and genotyping by reverse line blot hybridization assay. *Results*. CIN 2 regression rate was 49% (18/37), persistence as CIN 1 or CIN 2 was 22% (8/37), and progression to CIN 3 was 29% (11/37). Multiple HPV types were observed at admission in 41% (15/37) of cases. HPV 16 was detected at admission in 58% (11/19) of the cases that persisted/progressed and in 39% (7/18) of the cases that regressed. HPV 16 was considered possibly causal in 67% (10/15) of the cases that persisted or progressed and in 10% (1/10) of the cases that regressed (*P* = 0.01). *Conclusion*. Multiple HPV infections were frequently detected among women with CIN 2 at admission and during the followup. The CIN 2 associated with HPV 16 was more likely to persist or to progress to CIN 3.

## 1. Introduction

Cervical intraepithelial neoplasia (CIN) and cervical cancer derive from cellular transformations of the cervix epithelium after HPV infection. High-risk HPV (HR-HPV) persistent infections represent a necessary cause of cervical cancer, but not sufficient [1]. Additional conditions and events are required for the neoplastic transformation.

Firstly, it was thought that cervical squamous cell carcinomas (SCC) would always evolve from HPV infected normal cervical epithelium via a continuum, long-lasting consecutive CIN 1, CIN 2, and CIN 3 lesions [1]. However, it has been shown that clinically relevant CIN 2 or CIN 3 may be induced within 2-3 years following HPV high-risk infection, and another 10–12 years may pass until invasive cervical carcinoma would develop [2]. Most CIN 1 lesions that are associated with HR-HPV, and some CIN 2 lesions, should not be considered as true precursor stages of cervical cancer but rather the cytopathic effect of a productive viral infection [3]. Moreover, CIN 1 regression rate is high [4], as is the regression rate of CIN 2 [5–7], and both display viral expression patterns suggestive of productive viral infections. In contrast, some CIN 2 lesions and CIN 3 lesions show different viral gene expressions, leading to atypical proliferation and cell transformation. The development of CIN 3 arises in women who cannot resolve the HPV infection, and therefore the infections can persist for years or decades following initial exposure [8].

CIN 2 thus is a borderline lesion, can show clinical behavior similar to CIN 1 or to CIN 3, and occurs frequently in young women [6]. The 2012 Updated Consensus Guidelines of the American Society for Colposcopy and Cervical Pathology (ASCCP) [9] recommend that when a histological diagnosis of CIN 2 is specified for a young woman, observation is preferred, provided colposcopy is adequate.

The pattern of HPV infection and reinfection in women and the outcome of CIN 2 are not well known. A better discrimination of CIN 2 that trends to progression or regression could help in choosing the best clinical management. This study therefore aimed to describe the acquisition, persistence, and clearance of HPV infection in women with CIN 2 confirmed by biopsy followed for 12 months under expectant management.

## 2. Materials and Methods

### 2.1. Study Design and Ethical Methods

This cohort study was a part of a research for evaluating expectant management of women with CIN 1 and CIN 2, carried out between January 2007 and December 2009 at the State University of Campinas (UNICAMP), Campinas SP, Brazil. It was approved by the Institutional Review Board of the Faculty of Medical Sciences of UNICAMP.

### 2.2. Selection and Followup of the Women Studied

Women aged 18 to 46 years old were considered eligible for the study if they fulfilled the following criteria: (1) referral cervical smear showing atypical squamous cells of undetermined significance (ASCUS) or low-grade squamous intraepithelial lesion (LSIL); (2) biopsy showing CIN 2 reviewed by senior pathologist who was unaware of the DNA HPV test results [10]; (3) lesion completely visualized by colposcopy and squamocolumnar junction totally visible; (4) not being pregnant; (5) showing no evidence of any immunodeficiency diseases; (6) no history of previous invasive neoplasia; (7) HPV test result. The selection of patients was fully described in a previous publication (5). Fifty women consecutively fulfilled these criteria, agreed to participate in the study, and signed the informed consent.

The planned followup was visits every three months during one year with cervical smear, colposcopy, and sample collection for HPV detection. The colposcopic image showing a worsening of the lesion margem, color, and vascular pattern was submitted to biopsy. When the biopsy revealed CIN 2 or a less severe diagnosis, the woman was maintained in the follow-up plan. When the biopsy revealed CIN 3, immediate treatment by excision of the lesion was performed. At one year of followup, all the women who still showed cytological or colposcopy abnormalities were submitted to complete evaluation, and they were treated according to the final diagnosis. No ablative procedures were carried out.

During followup, 13 women were discontinued due to the following: five women diagnosed with persistence in the intermediate visit did not show up for the final visit; one woman tested positive for HIV; one woman missed three consecutive visits; one woman became pregnant; five women were without at least one HPV test during followup. After 12-month followup, 37 women had final diagnosis established.

### 2.3. Outcome of CIN 2 Followup

The final outcome of CIN 2 was classified as progression, persistence, or regression, according to the following criteria.

- Progression: biopsy showing CIN 3 detected at any time during the followup. No lesion worse than CIN 3 was revealed.
- Persistence: biopsy showing CIN 1 or CIN 2 at twelve-month followup.
- Regression: concomitant negative cervical smear and negative colposcopy or negative biopsy observed at any time during the followup and confirmed at twelve-month followup. If the patient had a HLSIL cervical smear and negative biopsy, it was not considered regression and the patient was submitted to excisional treatment.

### 2.4. Possible Causal HPV Type

The HPV type possibly associated with the CIN 2 lesion was considered the possibly causal type. For the CIN 2 lesions that regressed, possibly causal HPV was considered when HPV type was detected at admission and persisted up to the follow-up visit immediately before the regression. If the lesion persisted, either as CIN 1 or CIN 2, possibly causal HPV was considered when detected at admission and during the follow-up visits. Among the cases that progressed, possibly causal HPV was considered when detected at admission and at least until the follow-up visit when progression was detected. The possibly causal HPV type was considered undefined if the test was negative at admission or if it was not possible to associate the HPV type with the CIN 2 lesion as described previously.

### 2.5. Sample Processing and DNA Extraction

Aliquots of 200 *μ*L of Universal Collection Medium UCM (QIAGEN Sample and Assay Technologies, QIAGEN Biotechnology Brazil Ltda) were taken for polymerase chain reaction (PCR) testing and were centrifuged for 10 minutes at 13,000 g. The supernatants were immediately removed, and split cellular pellets were stored at −80°C before nucleic acid extraction and HPV detection. The cellular pellets were resuspended in 200 *μ*L of digestion solution (1 mM Tris, 200 mg of proteinase K/mL, 0.5% sodium dodecyl sulfate) and digested at 55°C for 2 hours. The digestion was followed by 5-minute incubation at 95°C to inactivate the proteinase K. Nucleic acids were purified by phenolchloroform extraction followed by ethanol precipitation. The DNA pellet was dried and was dissolved in 100 *μ*L of TE. Nucleic acids were stored at −80°C before HPV detection.

### 2.6. HPV Genotyping

HPV amplification and genotyping (HPV 16, 18, 26, 31, 33, 35, 39, 45, 51, 52, 55, 56, 58, 68, 6, 11, 40, 42, 53, 54, 57, and 66) were performed by the Roche Linear Array (LA) HPV genotyping test, according to manufacturer's instructions.

### 2.7. Data Analysis

Tables were constructed showing the HPV type at admission and for every three-month followup, grouped according to the clinical outcome. The association between two categorical variables was tested using Fisher's Exact Test. The cases with undefined HPV possibly causal type were not considered for the analysis.

## 3. Results

Table 1 shows the HPV detection at admission and every three-month follow-up visit. Women were grouped according to CIN 2 clinical outcome. Among the 37 women, 49% (18/37) regressed, 22% (8/37) persisted as CIN 1 or CIN 2, and 29% (11/37) progressed to CIN 3. There was no case of progression to invasive carcinoma detected during the study. At admission, 41% (15/37) had multiple HPV type detected and 11% (4/37) had negative HPV test. During followup, 54% (20/37) of women showed one or more of the new HPV types detected, resulting in eight more women with multiple HPV type detected.

Eighteen cases of CIN 2 regressed, of which 39% (7/18) had HPV 16 detected at admission, 16% (3/18) had HPV 56, and 16% (3/18) were HPV negative at admission. There were 33% (6/18) of the cases with multiple HPV types detected at admission and during followup 61% (11/18) presented new HPV type detected, one case with three new HPV types and five cases with two new HPV types. The HPV test was negative at admission or at least at one visit during followup in 61% (11/18) of cases. The HPV 16 was considered possibly causal in 6% (1/18) of cases.

Eight cases persisted, four as CIN 1, and four as CIN 2. At admission, HPV 16 was detected in 50% (4/8) of the cases that persisted, HPV 58 in 38% (3/8), and HPV test was negative in one case. Multiple HPV types were detected in 38% (3/8) of the cases at admission and new HPV types were detected in 50% (4/8). HPV 16 was considered possibly causal in 38% (3/8) of cases and HPV 58 in 38% (3/8).

Eleven cases (29%) progressed to CIN 3 up to twelve-month followup. HPV 16 was detected at admission in 64% (7/11) of the cases, HPV 52 in 27% (3/11), and HPV 68 in 18% (2/11). Multiple HPV types were detected at admission in 54% (6/11) of cases and new HPV type was detected during followup in 45% (5/11). Among the cases that progressed there was no negative HPV test. HPV 16 was considered possibly causal type in 64% (7/11) of cases.

Table 2 shows the association of HPV 16 with the CIN 2 clinical outcome. Cases that persisted as CIN 1 and CIN 2 were grouped together with the cases that progressed to CIN 3. HPV 16 was detected at admission in 58% (11/19) of the cases that persisted or progressed and in 39% (7/18) of the cases that regressed, but this difference was not statistically significant (*P* = 0.20). HPV 16 was considered possibly causal in 67% (10/15) of the cases that progressed or persisted and in 10% (1/10) of those that regressed, and the difference was statistically significant (*P* = 0.01). The cases with undefined HPV possibly causal type were not considered for the latter analysis.

## 4. Discussion

According to this cohort study, multiple HPV infections were frequently detected in women with CIN 2, as new HPV types were also frequently detected during twelve-month followup. The CIN 2 lesions associated with HPV 16 were more frequent among those lesions that progressed to CIN 3.

Cuschieri et al. [11] showed that multiple high-risk HPV infections were prevalent in young women, in high and low-grade cervical intraepithelial neoplasia, reflecting common sexual transmission of multiple high-risk HPV. Sometimes the lesion became HPV negative and at the next visit new infections were acquired. In this scenario, most of the HPV infections are probably productive, as pointed by Snijders et al. [1], that is, transitory infections, not inducing transformation process in the host cell.

Castle et al. [12] found that the presence of HPV 16 was positively associated with CIN 3 and they reported that CIN 2 caused by HPV 16 may be more likely to progress than CIN 2 caused by other high-risk HPV types [13]. Wentzensen et al. [14] also showed that CIN 2 related to HPV 16 was more likely to persist. The absolute risk of precancer diagnosis can approach 40% after 3–5 years of persistent HPV 16 infection [15]. Kjær et al. [16], in a population study, found that HPV 16 persistent infection was associated with high absolute risk for progression to high-grade cervical lesions.

HPV infection can be considered productive when the expression of viral gene products remains regulated, not leading to significant host cell changes. Transformation infections arise where the productive infection cannot be properly supported due to increased activity of viral protein E6 and E7, which leads to genomic instability in the infected cell, accumulation of oncogene mutations, further loss of cell-growth control, and ultimately precancer lesions and cancer [8, 17].

There was no negative HPV test in those cases that progressed, regardless of the HPV type. Among the eight cases that persisted, three cases presented negative HPV during followup, but in two cases the lesion persisted as CIN 1. Among the 18 cases that regressed, 11 presented negative HPV during followup, indicating transient infections. Only 4 out of 37 cases were HPV negative at admission, which could be a false negative or a true negative test, but none progressed.

The regression rate found is not different from the reported regression rate of CIN 2 in adult women, which ranges from 15 to 55% regression over 4–6 years of followup [13, 18]. Moscicki et al. reported CIN 2 regression rate of almost 70% among adolescents and young women and progression rate of 15% at three years [7]. This greater regression rate might be due to the younger age of the women studied (13 to 24 years old), which could reflect a shorter time of HPV persistent infection, and to the longer followup of three years. In the present study, the women's age was not used to select the population, the followup was twelve months, and the progression rate was similar to others that showed greater risk of CIN 2 progression when the lesion was associated with HPV 16 infection [1, 14].

This study has limitations due to small sample subject. The inclusion criteria were women with cervical smear referral showing ASCUS or LSIL, CIN 2 proven biopsy, and HPV test at admission and at least at one follow-up visit, which limited the number of selected women. Besides, multiple HPV infections were frequent in these women and, therefore, the indication of possibly casual HPV type for the CIN 2 was not always obvious. Despite the lack of reproducibility of CIN 2, the HPV types did not differ significantly between CIN 1, CIN 2, and CIN 3 [11]. False negative results with colposcopy and guided biopsy were expected, but this effect is minimized using cytology as cotesting.

For clinical practice, the findings of this study suggest that the HR-HPV testing would have limited utility to indicate those women with greater risk of CIN 2 progression since the rate of HR-HPV positive was high among women with CIN 2 regression. Nevertheless, expectant management, which may be proposed for young women, should be considered cautiously when HPV 16 infection is detected. In conclusion, infections with multiple HPV types were frequently detected at admission and follow-up visits as well as infections with new HPV types during followup. CIN 2 lesions with HPV 16 alone or in combination with other HR-HPV types are more prone to progression to CIN 3.

## Figures and Tables

**Table 1:** Distribution of HPV types at admission and every three months of followup according to clinical outcome of women with CIN 2.

Clinical outcome	Admission	Follow-up visit (month)				Possibly causal HPV
		3rd	6th	9th	12th	
Regression at 3rd	71	71, 58	Na	Neg	16	Undefined

Regression at 6th	16, 58	16	16	16	Na	58
	51	51	Neg	Neg	Neg	51
	26	Na	Neg	Na	16	26
	39, 52, 54, 56, 68	39, 52, 68	52, 51	58	Na	39
	16	16	16	16	16	Undefined
	31	31	31	31	Neg	Undefined
	Neg	Neg	Neg	Neg	16	Undefined
	16, 56, 66	56, 33	Na	33	Na	56
	Neg	16	58	Neg	Na	Undefined
	31	Na	61	Na	Na	Undefined

Regression at 9th	33	33, 16, 58	33	Neg	Neg	33
	16, 67	Na	67	Neg	16	67
	16	16, 39	Neg	Neg	Neg	16
	6, 16, 33	33	Neg	Neg	Neg	33
	39, 51, 53, 56	Na	39, 51	53, 18	39, 59	51

Regression at 12th	Neg	Na	Neg	Na	16, 58, 59	Undefined
	16	16	Na	Na	16	Undefined

Persistence as CIN 1	16	16	Na	16, 61, 81	16, 61, 81	16
	Neg	Neg	53	53	53, 16, 42	Undefined
	16	16	16	Neg	Na	Undefined
	81	51	81	16	Na	Undefined

Persistence as CIN 2	33	Na	Na	Na	33	33
	16, 58	16, 58	16, 58	16, 58	16, 58	16 or 58
	16, 58	16, 58	16, 58	16, 58	Na	16 or 58
	35, 58, 73	Neg	35, 58, 62	35, 58, 16	Na	35 or 58

Progression at 6th	16	16	16	16	Na	16
	16	16	16	16, 66	16, 66	16
	16, 33, 68	16, 33, 68	16, 33	16, 33	16, 33, 68	16 or 33

Progression at 9th	16, 68, 84	16, 84, 42, 58	16, 68, 58	16, 68, 58	16, 18	16

Progression at 12th	39	39	39	39	39	39
	16, 51, 52, 53	16, 52, 45, 84	16, 52, 84	16, 52, 58	16, 52	16 or 52
	16, 52	Na	Na	16, 52	16, 52	16 or 52
	52, 82	52, 82	52	Na	52	52
	6	6, 39	39	Na	Na	Undefined
	31	31	31	Na	Na	31
	16, 56	16, 56, 73	16, 56, 73	Na	Na	16 or 56

New HPV types detected during followup are underlined.

**Table 2:** Association between HPV 16 at admission and as possibly causal type and clinical outcome of CIN 2 during 12 months of followup.

HPV 16	Progression/persistence		Regression		*P* value
	*n*	%	*n*	%	
At admission					
Yes	11	58	7	39	
No	8	42	11	61	
Total	**19**	**100**	**18**	**100**	*P* = 0.20
Possibly causal					
Yes	10	67	1	10	
No	5	33	9	90	
Total	**15** ^ 1^	**100**	**10** ^ 2^	**100**	*P* = 0.01

^1^Four cases with undefined causal HPV.

^2^Eight cases with undefined causal HPV.
